# Primary Respiratory Bacterial Coinfections in Patients with COVID-19

**DOI:** 10.4269/ajtmh.20-0498

**Published:** 2020-06-03

**Authors:** Waqas Ahmed Chauhdary, Pui Lin Chong, Babu Ivan Mani, Rosmonaliza Asli, Riamiza Natalie Momin, Muhammad Syafiq Abdullah, Vui Heng Chong

**Affiliations:** Department of Medicine; PMMPHAMB Hospital; Tutong, Brunei Darussalam; National Isolation Centre; Tutong, Brunei Darussalam; Department of Medicine; RIPAS Hospital; Bandar Seri Begawan, Brunei Darussalam; Department of Medicine; PMMPHAMB Hospital; Tutong, Brunei Darussalam; National Isolation Centre; Tutong, Brunei Darussalam; Department of Medicine; RIPAS Hospital; Bandar Seri Begawan, Brunei Darussalam; Department of Medicine; PMMPHAMB Hospital; Tutong, Brunei Darussalam; National Isolation Centre; Tutong, Brunei Darussalam; Department of Medicine; RIPAS Hospital; Bandar Seri Begawan, Brunei Darussalam; E-mail: vuiheng.chong@moh.gov.bn

Dear Sir,

We read with interest the case report by Khaddour et al.,^1^ which reported a case of coinfection that led to delayed diagnosis of COVID-19. This case highlighted the importance of considering primary coinfection in patients with COVID-19.^1^ As the pandemic continues, the number of coinfections will increase. Not considering or testing for other respiratory pathogens can lead to delayed diagnosis that may lead to detrimental outcomes. We report our experience with bacterial respiratory coinfections in patients with COVID-19 with varying outcomes.

Of 141 confirmed cases of COVID-19 isolated and treated in Brunei Darussalam, five (3.5%) patients were found to have primary respiratory bacterial coinfections at different stages of illness (Table 1). Four were symptomatic, and one developed symptoms after admission. All had productive cough with purulent sputum. Only one case (Case 1) had complications (septic shock, and respiratory and renal failure) that required transfer to the intensive care unit. He eventually died of *Staphylococcus aureus* septicemia and COVID-19. Four patients were discharged after testing (reverse transcription-PCR) negative two consecutive times at least 24 hours apart.

**Figure 1.**
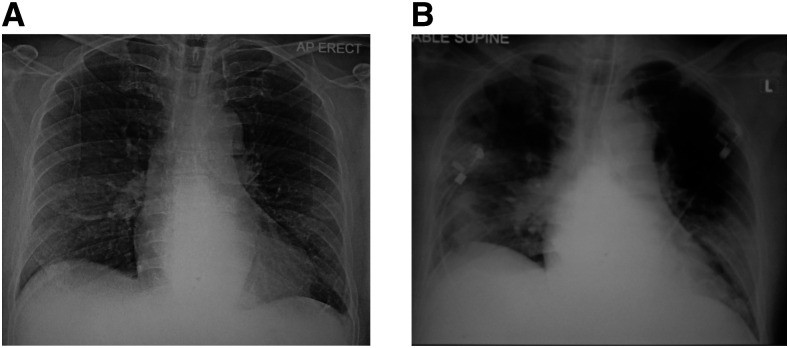
Chest radiographs. (**A**) Day 3 of hospitalization which was normal and (**B**) day 5 which showed bilateral consolidations.

**Table1:** Summary of demographic, investigation, and outcomes of patients

Case	Age (years)/gender	Source of COVID-19	Comorbid conditions	Symptoms at diagnosis	Coinfection pathogen	Sites	CXR (time of investigation)	Treatment (duration of treatment)	Outcomes (time of event)
1	64/M	Travel	Hypertension, dyslipidemia, thalassemia, and gout	Fever, chills, cough, and dyspnea (symptoms improved at admission)	*Staphylococcus aureus*	Blood sputum (+ve Gram stain) (day 1)	Normal (day 3) and consolidation bilaterally (day 5)	Oseltamivir (5 days), ciprofloxacin (3 days), lopinavir/ritonavir (11 days), hydroxychloroquine (5 days), piperacillin/tazobactam (7 days), and vancomycin (until death)	Septic shock, acute kidney injury, intensive care unit admission (day 4), intubation (day 4), dialysis (day 6), died of multi-organ failure, and septicemia (day 16)
2	61/M	Religious gathering	Hypertension, dyslipidemia, chronic constipation, and cervical spondylosis	Presymptomatic	*Klebsiella pneumonia* and methicillin-resistant *Staphylococcus aureus*	Sputum (day 1)	Normal (day-1), consolidation right side (day 7), and normal (day-15)	Oseltamivir (5 days), ceftriaxone (3 days), piperacillin/tazobactam (7 days), lopinavir/ritonavir (14 days)	Alive and discharged (day 17)
3	63/M	Religious gathering	Diabetes, dyslipidemia, Hypertension, and AF post-ablation	Fever, cough, and rhinorrhea	*Enterobacter gergoviae* and *Rothia mucilaginosa*	Sputum (day 1)	Normal (day 1)	Oseltamivir (5 days) and monitored and no treatment	Alive and discharged (day 24)
4	42/M	Travel	Nil	Fever, cough, dyspnea, rhinorrhea, and myalgia	*Streptococcus pneumoniae*	Sputum (day 1)	Normal (day 1)	Oseltamivir (5 days) and amoxicillin (7 days)	Alive and discharged (day 17)
5	29/F	Positive contact	Nil	Fever and cough	*Haemophilus influenzae*	Sputum (day 1)	Normal (day 2) and normal (day 6)	Oseltamivir (5 days), ceftriaxone (5 days), lopinavir/ritonavir (14 days)	Alive and discharged (day 22)

CXR = chest radiograph. Parentheses ( ) indicate the day of hospitalization when investigations (chest radiograph and sputum) were carried out, and outcome.

Although uncommon, primary pulmonary coinfection is increasingly being reported with COVID-19, especially with respiratory viruses.^1–5^ Nowak et al.^2^ reported respiratory viral coinfections of 3%. Zhu et al. reported higher rates of coinfection.^6^ This study tested 257 confirmed COVID-19 patients for respiratory pathogens and found 24 types of respiratory pathogens in 94.2%, with *Streptococcus pneumoniae*, *Klebsiella pneumoniae*, and *Haemophilus influenzae* as the most common pathogens. Most coinfections occurred within 1–4 days of presentation.^6^ Importantly, isolation of respiratory pathogens in sputum does not distinguish between colonization and clinically relevant infection. Coinfection with *Mycobacterium tuberculosis* has also been reported.^7^ Coinfections can result in diagnostic delay and less favorable outcomes. In the Khaddour et al.^1^ case, the diagnostic delay was due to an investigation protocol driven by limited access to tests for COVID-19. In our setting, we did not routinely test for other respiratory viruses, as there are no specific treatments apart from influenza virus, which was covered by our treatment protocol that included a 5-day course of oseltamivir. However, we did routinely screen for bacterial coinfections on admission. Had we not routinely screened our patients and instead followed a stepwise investigation protocol like Khaddour et al.,^1^ treatment would have been delayed and outcomes might have been different. Therefore, it is important to consider and screen for the possibility of coinfections with COVID-19.
